# Power in the flow: how menstrual experiences shape women's strength training performance

**DOI:** 10.3389/fspor.2025.1519825

**Published:** 2025-02-26

**Authors:** Sofia Ryman Augustsson, Anna Findhé-Malenica

**Affiliations:** Department of Sport Science, Faculty of Social Sciences, Linnaeus University, Kalmar, Sweden

**Keywords:** follicular phase, luteal phase, resistance training, physical performance, conventional content analysis

## Abstract

**Introduction:**

Hormone levels fluctuate significantly throughout the menstrual cycle (MC), potentially impacting physical performance during training. However, the number of studies examining women's experiences during strength training throughout the MC is limited. Therefore, the aim of this study was to explore women's perceptions of strength training during different MC phases.

**Methods:**

In this study, a qualitative study design was used where five women (24–32 years) with recreational experience in strength training kept an exercise diary during a MC. Data were collected using semi-structured interviews and analyzed using qualitative conventional content analysis, with an inductive approach.

**Results:**

From the analysis, three overarching themes describing the content of the interviews emerged: “Biopsychosocial Dynamics and Individual Variability in the Early Follicular Phase”, “From Peak to Breaking Point: Performance Dynamics from Late Follicular Phase to Ovulation” and “Diversity in Mental and Physical Well-Being During the Luteal Phase”. From women's perspectives, strength training performance seems to fluctuate across the different phases of the MC, influenced by both physiological and psychological challenges, though with individual variation.

**Discussion:**

The findings highlight the need for a holistic approach to managing the physiological and psychological challenges that may arise during each phase of the MC, along with the importance of social support. The results also stress that performance fluctuations across the MC are unique, further emphasizing the inability to recommend general phase-based exercise protocols.

## 1 Introduction

The menstrual cycle (MC) is a physiological rhythm essential for women's health (1). The cyclic pattern is driven by the endocrine interplay between hypothalamus—pituitary and ovaries and feedback regulation between hormones from these three structures (2). The MC consists of two main phases. The first phase, known as the follicular phase, begins on the first day of menstruation and lasts until ovulation, approximately 14 days (3). During this phase, gonadotropin releasing hormone stimulates the pituitary gland to release follicle-stimulating hormone (FSH), which stimulates the growth of ovarian follicles, one of which will mature into an egg (4). The concentrations of the ovarian hormone estradiol also rise, preparing the uterine lining for potential pregnancy. The peak of estrogen occurs a few days before ovulation followed by a short-term increase in gonadotropins, particularly luteinizing hormone (LH) surge triggers ovulation. After ovulation, LH and FSH levels decline, and the ruptured follicle forms the corpus luteum, which produces mainly progesterone and estrogen to a lesser extent to maintain the uterine lining for potential pregnancy (3). This is referred to as the luteal phase, the second phase of the MC, which begins after ovulation and lasts until menstruation, typically lasting 10–14 days (3). During this phase, if pregnancy does not occur, the corpus luteum disintegrates, progesterone and estrogen levels drop, and menstruation begins (2, 3). These hormonal changes, within the MC, can affect well-being by causing various physical and emotional changes (5). Psychological and physical symptoms are often intensified during the late luteal phase when estradiol and progesterone levels decline (6). These symptoms are commonly referred to as premenstrual syndrome (PMS).

PMS is a disorder which includes emotional, physical, and psychological symptoms that occur in the days or weeks leading up to menstruation affecting a significant proportion of women during their reproductive years (7, 8). Common symptoms include mood swings, bloating, fatigue, irritability, and breast tenderness (7) and emerging research highlights its impact on psychological well-being (9–11). Additionally, it has been suggested that PMS may contribute to a higher injury rate during the later luteal phase due to its impact on balance and neuromuscular function (8, 12, 13). Hence, physical performance often seems to be impaired during the late luteal together with the early follicular phases due to symptoms such as headaches, stomach and lower back pain or decreased motivation to exercise (7, 14–16).

Fluctuations in estrogen and progesterone during the MC are thought to impact exercise performance through various mechanisms. Estrogen may enhance muscle glycogen storage and fat utilization, while progesterone may have anti-estrogenic effects. Therefore, hormonal changes might cause performance variations. Research has also suggested that strength training during the late follicular phase results in greater increases in muscle strength (14, 17). During this phase, estrogen, which has anabolic effects, is the dominant hormone, with levels potentially rising from the mid-follicular phase (18). In contrast, progesterone, the dominant hormone in the luteal phase, is considered to have a catabolic effect (19). This, combined with the increase in testosterone around ovulation, suggests that strength performance may be higher during the first two weeks of the MC compared to the latter two (8, 20) but research has shown mixed results. Some studies have found no significant differences in strength performance across the MC (19, 21), while others have reported variations between the follicular and luteal phases (17, 22). It has been argued that the conflicting results from different studies may be due to the timing of the testing and when the term “follicular phase” is used, it seems unclear whether it implies to the early, mid, or late follicular phase (18). In a systematic review and meta-analysis, the largest performance decline was found between the early and late follicular phases of the MC, with the worst performance occurring in the early follicular phase (23). This may be because estrogen is low in the early but high in the late follicular phase (18). However, due to significant variability and inconsistent results in the literature, a personalized approach is recommended based on individual responses to exercise throughout the MC (23).

While most studies have used quantitative designs to investigate performance changes related to the MC (17, 19, 21, 22), the literature is scarce regarding women's subjective experiences of strength training performance across different MC. Studies investigating women's perceived performance during the MC have reported that hormonal fluctuations, especially variations in estrogen and progesterone, significantly affect exercise response and capacity (14–16, 24). However, most studies have focused on collegiate or elite athletes and examined perceived performance in specific sports or competitive contexts (14, 16). Moreover, existing studies have mostly focused on the physiological aspects of strength training performance in women. This paper proposes a single-subject approach to explore women's experiences and perceptions of physical performance during strength training in relation to the MC phases. Understanding these experiences could help set realistic goals for women in sports or rehabilitation. Such insights could be valuable for strength and conditioning coaches, as well as physical therapists. Given the variability in strength and physical performance across individuals (25, 26), a single-subject approach may offer a more nuanced perspective. Therefore, this study employs individual interviews using a qualitative, inductive approach. The aim of the study was to explore women's perceptions of strength training performance during different MC phases.

## 2 Methods

### 2.1 Study design and subjects

This study employed a qualitative interview design with an inductive approach to explore women's experiences with strength training during different phases of the MC. “Performance” in this context refers to subjects' perceptions of muscle strength, motivation, and energy during training sessions. Strength training is the use of resistance to muscular contraction to build the strength, anaerobic endurance and size of the skeletal muscles (27). The term “strength training” encompasses a wide range of training modalities, including body weight exercises, plyometrics (drop jumps, for example) and hill running (27). In this study, we included women that carried out strength training exercises at a gym. The selection of subjects was completed systematically through criterion selection, in order to include those who were suitable to participate (28). The inclusion criteria were women between 18 and 45 years who had at least one year experience with strength training and exercised at least twice a week. Additionally, subjects needed to track their MC using Natural Cycles (NC) (29) or in other ways measured basal body temperature (30). NC is a software, also available as a mobile app, designed for both contraception and fertility monitoring. The temperature is measured directly upon waking in the morning, before rising from bed. The reading is then recorded and entered into the app. By measuring basal body temperature with a basal body thermometer, the NC app estimates ovulation and predicts when her next period starts (29). Women using any form of hormonal contraception were excluded. Recruitment was conducted through social media. Initially, requests were shared in groups focused on tracking MC via basal body temperature. Subsequently, a female influencer, whose audience consisted of women interested in cycle tracking, posted the request on her platform. Seven women expressed interest in participating; however, two were excluded for not tracking their MC. Ultimately, five women were included in the study.

### 2.2 The interview guide

The authors developed a semi-structured interview guide consisting of questions addressing three main topics: (1) experiences of strength training; (2) experiences of the MC; and (3) experiences of the relationship between the MC and physical performance (e.g., strength, endurance, power, intensity and volume) during strength training. A pilot interview was conducted with one subject to test the guide and identify any potential shortcomings. Minor adjustments were made to the guide based on feedback from this pilot interview.

### 2.3 Procedure

Subjects were asked to maintain a training diary for one MC (approximately one month), recording their experiences from each training session in terms of strength (volume and intensity), perceived motivation, energy levels, and the current day of their cycle. This diary was intended to help them recall their experiences during the interviews. Data were collected through semi-structured interviews lasting approximately 30–45 min, conducted in the subjects' native language and later translated. The interviews were recorded via Zoom Video Communications, Inc. (password protected) and guided by the interview script. After each interview, one author (AFM) transcribed the recordings verbatim in a Word document. The interviews were then re-listened to for accuracy, enhancing the quality and reliability of the data.

### 2.4 Data analysis

The transcribed interviews were analyzed using qualitative conventional content analysis (31), given the scarcity of literature on women's experiences of performance in strength training throughout the different phases of the MC. This approach was chosen to authentically capture the women's emotions and provide a description of their lived realities in this specific context. The analysis process was inductive; the authors did not derive categories from existing theories or seek to validate them. Thus, no specific framework was employed, and the authors maintained no preconceived expectations for the data prior to analysis. Instead, they anticipated that themes related to strength training performance during various MC phases would emerge from the text (the interviews). The data processing followed these steps: (1) The transcribed interviews were read multiple times to give the authors an overall impression of the data, noting similarities and differences across interviews (32). (2) Meaning units were identified, which are the smallest segments containing insights relevant to the research question, consisting of related sentences or paragraphs (32). (3) These units were then condensed and labeled with codes, which facilitate the identification of concepts and capture meaningful aspects relevant to the research question (33). In this study, codes emerged purely inductively from the data. (4) The various codes were compared based on their differences and similarities and organized into categories describing the manifest content. (5) Finally, the categories were synthesized into overarching themes that captured the latent content of the interviews (31). These themes aimed to reveal common patterns across the categories and reflect underlying meaning. (6) The interviews were re-read for reflection to ensure that the themes aligned with the study's purpose and that the meaning units were accurately represented. Throughout the analysis process, the authors engaged in open and critical discussions at each step until consensus was reached (33). Examples of this analytical process are presented in the supplementary file (Supplementary Table S1).

## 3 Results

Five women between 24 and 32 years participated in the study. All subjects participated in recreational strength training 2–3 times a week on recreation level and had a regular MC, which they mapped by measuring basal body temperature as described. Based on the purpose of the study, three themes and seven categories emerged in the analysis, (Table 1). All subjects reported both mental and physical symptoms related with the MC which affected training.

**Table 1:** Themes and categories from the analysis.

Themes	Categories
Biopsychosocial Dynamics and Individual Variability in the Early Follicular Phase	Motivated and strong
	Reduced motivation and pain
	Social support
From Peak to Breaking Point: Performance Dynamics from Late Follicular Phase to Ovulation	Reaching the limit
	Breaking point at ovulation
Diversity in Mental and Physical Well-Being During the Luteal Phase	Reduced motivation, weakness and exhaustion
	Feeling strong and energized

### 3.1 Theme 1. Biopsychosocial dynamics and individual variability in the early follicular phase

This theme describes the experiences of the early follicular phase. Subjects described varying mental and physical experiences in relation to their strength training. The theme emerged from three categories: “Motivated and strong”, “Reduced motivation and pain” and “Social support”. Most subjects in the study reported feeling strong and energized during the early follicular phase, specifically a few days into their period or just after it ended. Two subjects described a renewed sense of energy once menstruation began, noting that training became easier a few days into their period. Subjects also noted that their motivation was higher at the early follicular phase compared to the end.

‘I exercised, I still had some periods, but I still felt strong and resilient’ (No. 1)

‘….but already day 2, 3, 4 I feel..very social and happy’ (No. 4)

‘Once the period has arrived, at least after day 1–2, I feel the energy increase. Training is much easier, I'm a bit more excited about training’ (No.5)

‘I feel much more motivated at the beginning of my cycle’ (No. 1)

Still, not all training days in the early follicular phase were experienced as carefree. Some subjects did lack motivation during some days due to menstruation pain. One subject felt unmotivated to exercise during menstruation, attributing her fatigue and lack of motivation to menstrual pain. She also expressed the need of encouragement from others to cope and to perform which emphasizes the importance of social support. Another subject mentioned being more prone to injury during menstruation because her body struggled to keep up.

‘Motivation level varies a bit with the cycle, so that when you have pain because you have menstrual pain, it is very tired..it is much harder to go and practice’ (No. 3)

‘I still felt that if I didn't have the push from the others (training mates), I probably wouldn't have had the same strength’ (No.2)

‘If I hurt myself, I often do it when I have my period…. I have noticed’ (No. 3)

### 3.2 Theme 2. From peak to breaking point: performance dynamics from late follicular phase to ovulation

This theme explores the subjects' experiences with strength training during the late follicular phase, leading up to and including ovulation and were shaped from two categories; “Reaching the limit” and “Breaking point at ovulation”. All subjects indicated that the late follicular phase was the time when they felt the most energy and motivation for strength training. They reported feeling their strongest and most powerful during this period.

“. so I left the session and just oh, how strong I felt today! Now almost did a personal best here” (No. 3)

“Yes, but life gets a little easier in general, the closer you are to ovulation. You have more energy, you're more excited and happy and yes, it’s a bit easier. and you can handle both the workload and things outside work. You perform at your best in everything, I would say” (No. 5)

The theme also describes the subjects' experiences of clear breaking point in their strength training performance between the follicular and luteal phases, specifically at their estimated ovulation time. Subjects felt stronger and performed better before ovulation, but noticed a decline in performance afterward, experiencing weakness and a lack of energy.

“There is a very strong like breaking point when I ovulated. Because before that, it felt great and I felt strong,.. and then after ovulation I have written pain, it starts to feel tiring, hard to… I could not cope” (No. 3)

### 3.3 Theme 3. Diversity in mental and physical well-being during the luteal phase

The MC phase perceived as the most negative was the late luteal phase. Subjects described a range of mental and physical experiences related to their performance in strength training during this phase. Still, some subjects experienced increased strength 2–3 days before their period. To describe this, two categories were formed; “Reduced motivation, weakness and exhaustion” and “Feeling strong and energized”. All subjects reported experiences of reduced training energy during the luteal phase, along with feelings of weakness and a greater need for recovery time. Subjects also reported feeling unmotivated to exercise in the days leading up to menstruation.

“Tired and….that I'm running out of energy” (No 2)

“Just after ovulation I feel my mood dropping….I take a few kilos off “ (No. 4)

God, what a big difference in weight, I can't even lift…” (No. 4)

“It requires more time to recover towards the end of the cycle… it wears me out” (No. 5)

One subject felt very emotional during this phase, which she believed could negatively impact her training.

“Very sensitive, psychologically, and I think it makes the training less effective” (No 1)

Although the subjects experienced fatigue in the days before their period, they still felt strong and some very strong 2–3 days before their period.

“At that time I was very close to a personal best in Power Cleans. I felt strong” (No. 2)

“ .. it just feels heavy and boring just before my period” (No. 4)

“Day 22, it was explosive strength again. I felt, well, the weights came up, but the motivation wasn't as high.. the energy level was quite low” (No. 5)

## 4 Discussion

This study used a qualitative, inductive approach to explore women's experiences and perceptions of strength training performance during different phases of the MC. From the interviews, three main themes emerged, highlighting the varying physical and mental experiences associated with strength training in women throughout the different phases of the MC. The results of this study underscore the complex interplay between the MC, training, and performance.

### 4.1 Theme 1. Biopsychosocial dynamics and individual variability in the early follicular phase

The follicular phase was identified as a time when strength training performance varied, with several subjects noting a lack of energy during the initial days of menstruation. According to previous studies (14–16), lack of motivation and fatigue may be attributed to PMS that typically subsides with the onset of menstruation. This may explain why subjects reported feeling unmotivated to engage in strength training during the first 1–3 days of their period. In addition, subjects described being more prone to injury during menstruation. This may be attributed to increased fatigue during the early follicular phase, which could also impact neuromuscular function (34, 35). Nevertheless, since we did not assess the incidence of injuries, we cannot ascertain whether injuries occurred or determine their cause. Moreover, the lack of motivation was associated to menstruation pain according to some subjects which clearly stresses the interconnection between mental and physical well-being. The expressed need for support from training peers further highlights the importance of a biopsychosocial approach to understanding health and training performance in relation to the MC. The MC has been previously cited as an ideal example of a biopsychosocial phenomenon, being a natural physiological process that both influences and is influenced by women's behavior (36). The findings of this study support existing knowledge and provide new data on the phenomenon as it relates to strength training experience. Still, subjects also described experiences of increased energy a few days into menstruation during the early follicular phase. A renewed sense of strength, performance, and energy was described, along with a refreshed motivation to engage in strength training. This new motivation and energy could be caused by the hormones Estrogen, FSH and LH that gradually increases during the follicular phase (20, 37, 38). Thus, the women in this study experienced both positive and negative effects related to menstruation, highlighting individual variability and the inability to recommend general phase-based exercise protocols. Instead, the varied experiences observed in this study support the use of previously recommended personalized training methods throughout the MC (23).

### 4.2 Theme 2. From peak to breaking point: performance dynamics from late follicular phase to ovulation

In the present study, most subjects reported increased perceived energy and strength at the late follicular phase. Around ovulation, subjects felt their performance was at its peak, describing themselves as especially strong, motivated, and full of energy. This may be attributed to the peak in anabolic hormones during ovulation (8, 20). Previous research indicates that strength performance varies across different phases of the MC (17, 22) with muscle strength generally higher during the follicular phase compared to the luteal phase, consistent with the experiences described by the women in this study. However, as no direct strength measurements were taken, definitive conclusions about strength fluctuations cannot be drawn. The perceived changes in strength noted here may be more related to mood and motivation than to actual physical strength gains. Nonetheless, perceived impacts on energy and motivation, such as feeling more motivated and energized for strength training, can influence physical performance (39, 40). Enhanced well-being and motivation during the late follicular phase may also contribute to the subjective experience of greater muscular strength.

Subjects identified a breaking point around their estimated ovulation time. For two subjects, this point was particularly distinct, as they experienced a notable decline in strength, motivation, and energy following ovulation. Another subject reported that while their strength remained stable, their energy levels dropped. This breaking point aligns with the transition from the follicular phase to the luteal phase, during which estrogen, FSH, and LH levels decrease rapidly after ovulation (8). This hormonal shift may explain the perceived reduction in strength and energy.

### 4.3 Theme 3. Diversity in mental and physical well-being during the luteal phase

In the present study, subjects reported the luteal phase as the period during which their strength training performance was at its lowest. Many described a noticeable drop in energy levels following ovulation, accompanied by physical symptoms such as nausea, fatigue, weakness, and a need for longer recovery times. Perceptions of performance also varied during the later part of the luteal phase, with subjects describing their training as heavy and boring. Most reported a lack of motivation and reluctance to train. Previous studies have noted that numerous women experience various PMS symptoms during this phase, such as low motivation and physical discomfort (14–16, 41). Previous research has also highlighted fatigue as a prevalent and debilitating aspect of PMS (42). Hormonal changes, particularly variations in estrogen and progesterone levels, may impact neurotransmitter regulation and exacerbate PMS symptoms such as fatigue and reduced strength. Progesterone, the predominant hormone during the luteal phase, is catabolic and can inhibit force development, which may help explain the weakness reported by several subjects (14, 19, 20). Additionally, the psychological toll of managing PMS symptoms may further intensify feelings of fatigue. Some subjects reported psychological symptoms that they felt negatively impacted their training, describing feelings of anxiety, increased psychological sensitivity, lack of motivation, and even depression. Whether experiencing psychological or physical symptoms, most subjects reported a reduced ability to train as intensively as during ovulation and/or a tendency to tire more quickly. These findings underscore the importance of a holistic approach to understanding and managing both the physiological and psychological aspects of PMS. Future research should investigate the underlying mechanisms further and explore targeted interventions to mitigate the effects of PMS on motivation and fatigue in training contexts.

Some subjects noted that despite feeling fatigued, they actually felt strong in the days leading up to menstruation. One subject mentioned achieving a personal best in a training session two days before her period began. Another reported that her overall strength remained consistent throughout her cycle, although she experienced low motivation, lack of energy, and a need for longer recovery. These variations suggest that experiences during the luteal phase differ, potentially due to PMS impacting individuals differently. It's also possible that some subjects choose to push through symptoms and maintain their usual routines, while others adjusted their activity levels accordingly. The findings emphasize the importance of recognizing that performance fluctuations across the MC are unique and further stress the difficulty of recommending general phase-based training. For coaches and physical therapists working in strength training, understanding the diverse effects of the MC on women's experiences can be a valuable tool for supporting and motivating athletes to perform, regardless of their energy levels.

### 4.4 Methodological considerations

A qualitative inductive approach was chosen, with data analyzed through conventional qualitative content analysis. This approach allows the authors to draw information directly from the subjects, without imposing predetermined theoretical frameworks. As a result, the findings are rooted entirely in the subjects' unique perspectives and experiences, making this method particularly useful for gaining insight when no underlying theories are in place (31, 32). Subjects were selected strategically (28) with the sample limited to women with regular MC to ensure reliable results. Although the sample was relatively small (five women), sample size in qualitative research is often less critical than the richness and variation of the data generated (32). As Patton suggests, sample size in qualitative studies is chosen purposefully to capture information-rich cases, enabling in-depth exploration of experiences and meanings (28). This study prioritized internal generalization—providing detailed insights within this specific context—rather than generalizing findings to other settings, groups, or populations (external generalization) (43). To ensure internal validity, we adhered to criteria for dependability, credibility, and transferability (32).

One potential limitation is that observing a single MC may not fully capture the variability or longitudinal changes in experiences, which could limit the findings' validity. To mitigate this, subjects maintained a training diary throughout the cycle to help accurately recall their experiences and reduce the risk of inaccurate reporting. To further increase validity, both authors conducted the analysis independently before discussing results and reaching consensus (44). Every step of the analysis underwent critical review within the research team to ensure accuracy and alignment. The authors' personal experience with strength training was considered in interpreting the data, adding contextual understanding. Finally, while basal body temperature measurements were used to assess each subject's MC, we acknowledge potential limitations with this method. The pre-ovulatory LH surge and blood hormone levels were not measured, so ovulation could not be confirmed. Nonetheless, we believe that subjects were well attuned to their monthly physical changes, which are typically unmistakable. Women using any form of hormonal contraception were excluded to avoid interference with the natural MC experience (45).

## 5 Conclusions

From women's perspectives, strength training performance appears to fluctuate across the different phases of the MC, though individual experiences vary. This stresses the importance of understanding one's unique cycle to optimize training and that a training diary, including documentation of MC, could be valuable. Furthermore, the findings emphasize the need for a holistic approach to managing both physiological and psychological challenges that may arise during each phase of the MC and the importance of social support. Strength and conditioning coaches, as well as physical therapists, may benefit from being aware of these individual variations in physical and mental experiences and their effects on performance. Future research could focus on developing personalized approaches to enhance strength performance in both sports and rehabilitation settings for women.

## Data Availability

The raw data supporting the conclusions of this article are not publicly available due to ethical restrictions.
