# Supplementary material for: Power in the flow: how menstrual experiences shape women's strength training performance
Source: Front Sports Act Living. 2025 Feb 26;7:1519825. doi: 10.3389/fspor.2025.1519825 (PMC11897035; doi:10.3389/fspor.2025.1519825)
Supplement: Supplementary file 1 [file Table1.docx]

Supplementary Material

# Supplementary Data

**Table 1.** Examples of meaning units, condensed meaning units, codes, categories and themes from the present data.

| **Theme** | **Category** | **Code** | **Condensed meaning unit** | **Meaning unit** |
| --- | --- | --- | --- | --- |
| **Biopsychosocial Dynamics and Individual Variability in the Early Follicular Phase** | Motivated and strong | Strong and resilient | I felt strong and resilient  despite having period | The next time I worked out  I still had little period, but felt  strong and resilient |
|  |  | Joyful | I did a little extra because it felt great | After I practiced today.. so just like this with God I run a little extra, I think it's a little extra fun.. feels great for the body |
|  |  | Motivated | More motivated at the beginning | Much more motivated at the beginning of my cycle |
|  | Reduced motivation and pain | Injuries | Hurts me more often during menstruation | If I hurt myself, I often do it when I have my period…I have noticed |
|  |  | Unmotivated | I am unmotivated  to exercise during my period | Very unmotivated to go and work out when I have my period |
|  | Social support | Friends | Push from the others | I still felt that if I didn't have the push from the others, I probably wouldn't have  had the same strength |
| **From Peak to Breaking Point: Performance Dynamics from Late Follicular Phase to Ovulation** | Reaching the limit | Peak performance | I left the session feeling really strong, almost  doing a personal best | .. so I left the session and just oh, how strong I felt today! Now I've kind of almost run a personal best here |
|  |  | Incredible | Being completely incredible | Then I actually got on cycle day 24 then it was actually confirmed…  I ovulated later .. and then I was really really strong. Being completely incredible! |
|  | Breaking point at ovulation | Changes | I need more rest between sets and after the session compared to before ovulation when I had more energy | I need more rest between sets and afterwards I cannot train even if someone asked me the day after, while there, before and around ovulation, I could so easily train the day after without problems I think.. |
|  |  | Breaking point | Strong breaking point when ovulating | There is a very strong breaking point when I ovulated |
| **Diversity in**  **Mental and Physical Well-Being During the Luteal Phase** | Reduced motivation, weakness and exhaustion | Tired and exhausted | I feel sick and it takes longer to recover | I feel sick after the session, it takes a little longer before I feel recovered |
|  |  | Heavy and boring | It feels heavy and boring before my period | ….it just feels heavy and boring just before my period has come |
|  | Feeling strong and energized | Fighting fatigue | I felt strong and could push through everything even though it was hard | Really strong even though it was hard .. I still felt I could do everything and could push more in everything. I felt sick,  but I managed |
